# Synthetic interpolated DSA for radiation exposure reduction via gamma variate contrast flow modeling: a retrospective cohort study

**DOI:** 10.1186/s41747-023-00404-2

**Published:** 2024-02-16

**Authors:** Andrew Abumoussa, Alex Flores, Christiana M. Cornea, Diwash Thapa, Amy Petty, Aaron Gelinne, Scott Elton, Carolyn Quinsey, Deanna Sasaki-Adams, Sten Solander, James Ho, Edward Yap, Yueh Z. Lee

**Affiliations:** 1 Department of Neurosurgery, UNC School of Medicine, Chapel Hill, NC 27516 USA; 2 Department of Neurosurgery, Baylor College of Medicine, Houston, TX 77030 USA; 3 Department of Neurosurgery, University of Cincinnati College of Medicine, Cincinnati, OH 45267 USA; 4 Department of Radiology, UT Southwestern Medical Center, Dallas, TX 75390 USA; 5 Department of Dermatology - Duke University, Durham, NC 27710 USA; 6 Department of Neurosurgery, University of Arkansas for Medical Sciences, Little Rock, AR 72205 USA; 7 Department of Radiology, UNC School of Medicine, Chapel Hill, NC 27516 USA; 8 Department of Neurology, UNC School of Medicine, Chapel Hill, NC 27516 USA; 9 Department of Biomedical Engineering, University of North Carolina at Chapel Hill, Chapel Hill, NC 27514 USA; 10 Department of Physics and Astronomy, University of North Carolina at Chapel Hill, Chapel Hill, NC 27599 USA

**Keywords:** Angiography (digital subtraction), Contrast media, Radiation protection, Radiology (interventional), Retrospective studies

## Abstract

**Background:**

Digital subtraction angiography (DSA) yields high cumulative radiation dosages (RD) delivered to patients. We present a temporal interpolation of low frame rate angiograms as a method to reduce cumulative RDs.

**Methods:**

Patients undergoing interventional evaluation and treatment of cerebrovascular vasospasm following subarachnoid hemorrhage were retrospectively identified. DSAs containing pre- and post-intervention runs capturing the full arterial, capillary, and venous phases with at least 16 frames each were selected. Frame rate reduction (FRR) of the original DSAs was performed to 50%, 66%, and 75% of the original frame rate. Missing frames were regenerated by sampling a gamma variate model (GVM) fit to the contrast response curves to the reduced data. A formal reader study was performed to assess the diagnostic accuracy of the “synthetic” studies (sDSA) compared to the original DSA.

**Results:**

Thirty-eight studies met inclusion criteria (average RD 1,361.9 mGy). Seven were excluded for differing views, magnifications, or motion. GVMs fit to 50%, 66%, and 75% FRR studies demonstrated average voxel errors of 2.0 ± 2.5% (mean ± standard deviation), 6.5 ± 1.5%, and 27 ± 2%, respectively for anteroposterior projections, 2.0 ± 2.2%, 15.0 ± 3.1%, and 14.8 ± 13.0% for lateral projections, respectively. Reconstructions took 0.51 s/study. Reader studies demonstrated an average rating of 12.8 (95% CI 12.3−13.3) for 75% FRR, 12.7 (12.2−13.2) for 66% FRR and 12.0 (11.5−12.5) for 50% FRR using Subjective Image Grading Scale. Kendall’s coefficient of concordance resulted in *W* = 0.506.

**Conclusion:**

FRR by 75% combined with GVM reconstruction does not compromise diagnostic quality for the assessment of cerebral vasculature.

**Relevance statement:**

Using this novel algorithm, it is possible to reduce the frame rate of DSA by as much as 75%, with a proportional reduction in radiation exposure, without degrading imaging quality.

**Key points:**

• DSA delivers some of the highest doses of radiation to patients.

• Frame rate reduction (FRR) was combined with bolus tracking to interpolate intermediate frames.

• This technique provided a 75% FRR with preservation of diagnostic utility as graded by a formal reader study for cerebral angiography performed for the evaluation of cerebral vasospasm.

• This approach can be applied to other types of angiography studies.

**Graphical Abstract:**

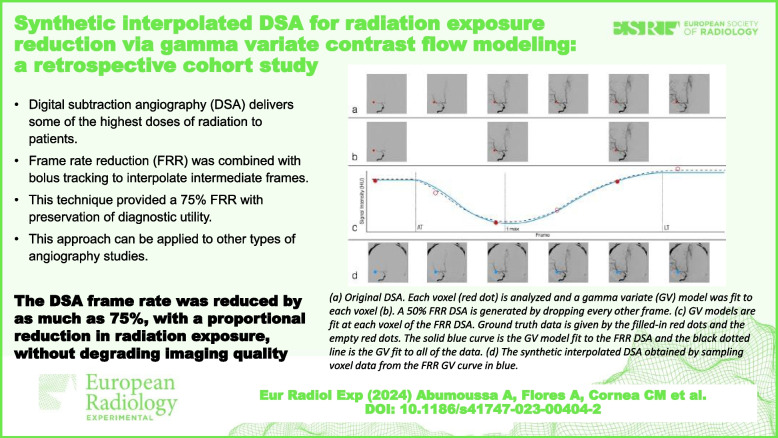

**Supplementary Information:**

The online version contains supplementary material available at 10.1186/s41747-023-00404-2.

## Background

Digital subtraction angiography (DSA) is the standard of care for the evaluation of several vascular pathologies. Compared to other modalities such as ultrasound, computed tomography (CT), and magnetic resonance (MR) angiography, DSA’s ability to both assess and provide direct vascular intervention is unique. One major pitfall of DSA is the cumulative radiation dosage (RD) delivered to patients. For standard diagnostic cerebral angiography phantom studies estimate the average radiation dosages to be 2.71 mSv, almost five times the dose of a head CT angiography [1]. 

Given the high RD delivered, it becomes critical that DSA acquisition protocols be optimized to minimize cumulative RD. Factors such as patient size, weight, and imaging system’s hardware are non-modifiable determinants of the total RD delivered. Modifiable factors are the image acquisition parameters, specifically, the frame rate and imaging flux. Angiographic dose reduction can be achieved by either reducing the number of frames acquired or adjusting the power settings per frame: voltage to change acceleration of the x-ray photons to adjust penetration energy, or current to modify the photon flux.

Dosimetry studies demonstrate that RD is directly proportional to frame rate during acquisition [2]. This is leveraged with neuro-interventional radiology as acquisition protocols reduce frame rate during capillary and venous phases, the phases with the most imaging redundancy [3]. Reducing the energy delivered per frame requires changing the factory presets on the imaging hardware (necessitating vendor support from radiation physicists) and degrades imaging quality. This, in turn, requires further postprocessing in the form of automatic voxel shift, temporal averaging of consecutive frames to suppress uncorrelated quantum noise and a multiphasic spatial filter that selectively smooths the images to recover fidelity [4]. This technique was evaluated and found capable of reducing RD by 61% per frame during endovascular aneurysm repairs [5]. Studies assessing DSA image quality with respect to frame rate or energy reduction are missing in the literature.

This work aims to evaluate how far DSA frame rates can be reduced while preserving a study’s diagnostic utility. We propose a post-processing technique which is independent of a DSA underlying hardware system. We leverage contrast flow through parenchyma with gamma variate (GV) models, like multimodal perfusion imaging in CT perfusion, dynamic susceptibility contrast MR perfusion, and more recently DSA perfusion [6–16]. We hypothesize that by using GV models, DSA frame rates can be deliberately reduced, and then resynthesized, without compromise to the overall image’s diagnostic utility.

## Methods

### Patient demographics

Our experiments were set up to evaluate whether frame rate reduction (FRR) obtained through postprocessing could preserve the anatomic detail required for clinical decision-making and post-therapeutic evaluations. FRR with post-processing frame interpolation can be applied to any DSA study. This work focused on cerebral angiography due to the high RD delivered when these studies are performed and to minimize the confounding effects of motion artifact that occurs during respirations as observed in the chest or abdominal DSA.

This retrospective study was approved by the UNC Institutional Review Board committee with a waiver of consent granted. A consecutive set of cerebral angiographic studies performed for the treatment of cerebral vasospasm following aneurysmal subarachnoid hemorrhages was evaluated. All DSAs were performed by 4 board-certified interventionalists, obtained consecutively at a single stroke-certified, academic institution. Inclusion criteria were (1) DSA included one diagnostic series with acquisition of the arterial, capillary, and venous phase; (2) DSA included one post-therapeutic series with acquisition of the arterial, capillary, and venous phase; and (3) the DSA series included 16 or more acquired frames. Exclusion criteria were (1) significant patient motion during the DSA acquisition for all series; and (2) significant changes in magnification or angles of DSA acquisition between diagnostic and post-therapeutic run. We retrospectively identified 38 consecutive patients that met the inclusion criteria between 2004 and 2020.

### Data acquisition

DSA acquisition was performed with biplane angiography equipment (Axiom Artis Siemens AG, Erlangen, Germany). Data was anonymized and transferred offline for analysis. All postprocessing was performed on raw DSA images using code written in Java (Oracle Corporation, Austin, TX, USA) as an extension in the MIM platform (MIM Software Inc, Beachwood, OH, USA).

### Experimental design

**Fig. 1:**
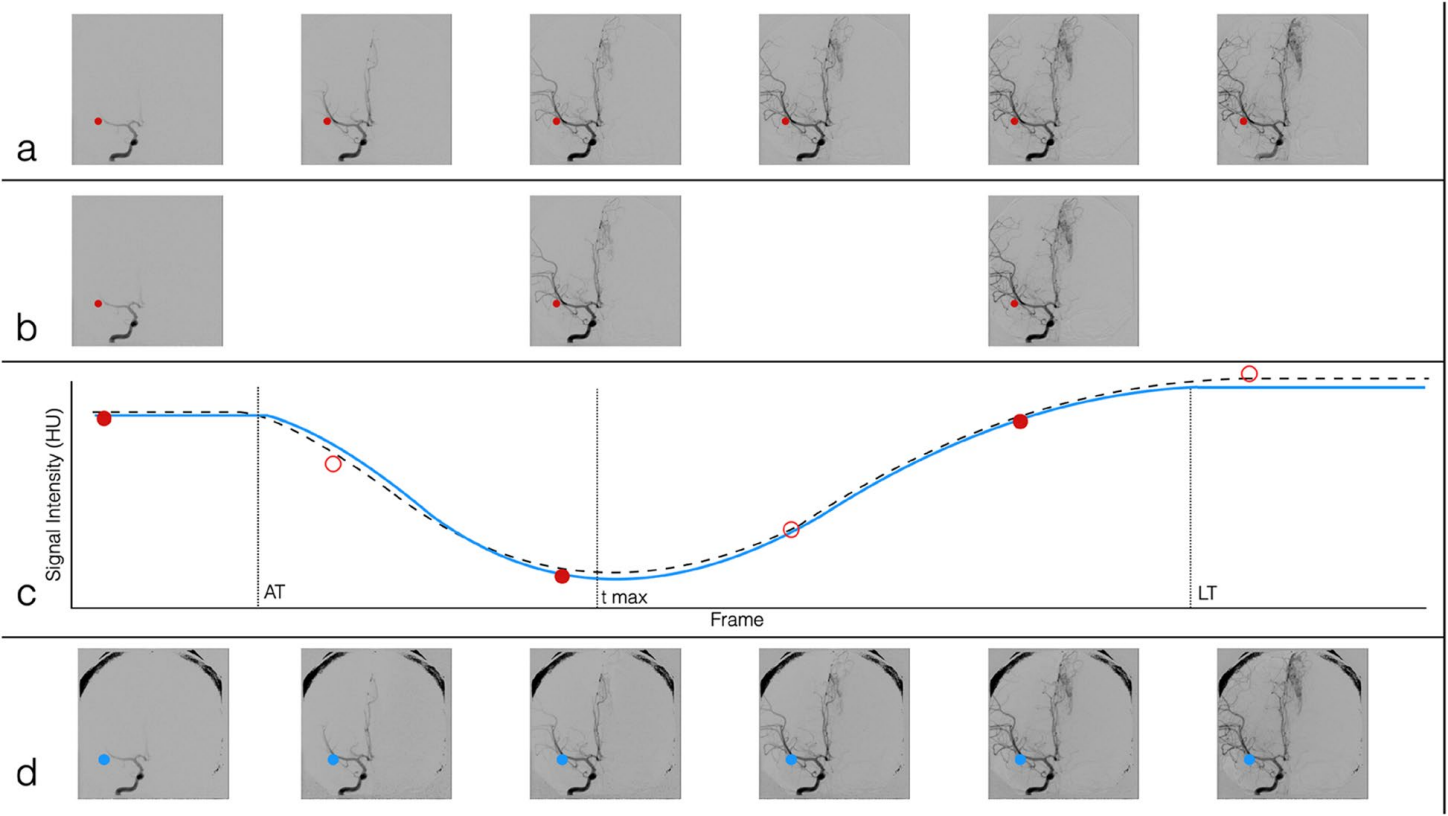
Graphical representation of the experimental DSA FRR. **a** Original DSA with all frames represented. Each voxel (represented by the red dot) is analyzed and a GV model was fit to each voxel during the processing. **b** A 50% FRR DSA is generated by dropping every other frame (a 66% FRR would drop 2 out of every 3 frames, and a 75% FRR is generated by dropping 3 out of 4 frames). **c** GV models are fit at each voxel of the FRR DSA. Ground truth data is comprised of both the filled-in red dots and the empty red dots. The solid blue curve is the GV model fit to the FRR DSA and the black dotted line is the GV fit to all of the data. **d** The sDSA obtained by sampling voxel data from the FRR GV curve in blue. *AT* Arrival time, *DSA* Digital subtraction angiography, *FRR* Frame rate reduction, *GV* Gamma-variate, *LT* Leave time, *sDSA* Synthetic DSA, *Tmax* Time at which maximal signal intensity is observed

Anteroposterior and lateral series were obtained for all subjects that met the criteria for inclusion in the study. FRR was experimentally performed by dropping the original acquired frames to reach predefined FRRs of 50%, 66%, and 75%. GV functions were fit to each voxel intensity-time series for each FRR DSA. The mathematical reasoning and algorithmic steps are provided in the Additional file 1. The GV functions from the FRR DSAs were then sampled at the original frame rate to create a synthetic DSA (sDSA) at each simulated FRR (Fig. 1a–d).

Errors for each sDSA frame were automatically evaluated, comparing the sDSA voxel data to the ground truth DSA was performed for all sDSA studies. The average percent error of the intensity at each voxel over the entire study was calculated and rendered as heatmaps to show the spatial distribution of the errors. Errors were calculated as the norm of the difference between the synthesized and the ground truth data using the following formula^1^:

1 $${R}_{SI}\left(t\right)=\frac{\left|{SI}_{ij}^{o}(t)-{SI}_{ij}^{s}(t)\right|}{{SI}_{ij}^{o}(t)}$$

where ${R}_{SI}\left(t\right)$ is the error in signal intensity of the sDSA at time $t$, ${SI}_{ij}^{o}(t)$ is the signal intensity of the voxel at $i,j$ of the original DSA, and ${SI}_{ij}^{s}(t)$ is the signal intensity of the voxel at the same location of the sDSA [6, 9, 16, 17].

### Reader study

A reader study was then performed on a subset of the patients to evaluate the diagnostic value of the sDSA when directly compared to the original study. Readers were blinded to the FRR levels, and the sDSA/DSA pairs were randomized and presented to the readers for evaluation. The original DSA, serving as a reference study, was displayed on one monitor (left) and the corresponding sDSA at a random FRR level was displayed on an adjacent monitor (right). The order of presented pairs was randomized.

To sufficiently power this study using an alpha error of 0.05, a power of 0.75 for our continuous variable (Radiologist grading scale, Table 1) with a hypothesized mean and standard deviation of 16 ± 2 for a 50% FRR and 13 ± 3 for 66% FRR, 12 patients were required. To provide as large of a sample from our study population as possible, the decision was made to include 12 diagnostic and 12 post therapeutic scans, each from different patients of the original 31 patients identified by our inclusion criteria. Therefore, 48 angiographic runs were evaluated (48 angiographic runs representing 24 sets of anteroposterior and lateral projections from 24 randomly selected patients). 50% of the DSAs were diagnostic angiograms and 50% of the DSAs were post-therapeutic angiograms).

**Table 1:** Reader study scoring rubric

Score	Description
*Arterial phase* 1. Uninterpretable 2. Severely deficient but interpretable 3. Moderately deficient but interpretable 4. Mildly deficient but interpretable 5. Perfect reproduction of reference	*Large and small arteries visible and crossing arteries are distinct* 1. Unusable for diagnosis 2. Small vessels not discernible; larger vessels not sharply defined 3. Fair vessel delineation; useful for diagnosis 4. Good vessel definition; small vessels visible 5. Excellent visualization of proximal through small distal vessels
*Capillary phase* 1. Uninterpretable 2. Severely deficient but interpretable 3. Moderately deficient but interpretable 4. Mildly deficient but interpretable 5. Perfect reproduction of reference	*Capillary blush — assessment of contrast flow through capillary vessels* 1. Unusable for diagnosis 2. Small vessels not discernible; larger vessels not sharply defined 3. Fair vessel delineation; useful for diagnosis 4. Good vessel definition; small vessels visible 5. Excellent visualization of proximal through small distal vessels
*Venous phase* 1. Uninterpretable 2. Severely deficient but interpretable 3. Moderately deficient but interpretable 4. Mildly deficient but interpretable 5. Perfect reproduction of reference	*Venous vasculature visible and distinct* 1. Unusable for diagnosis 2. Small vessels not discernible; larger vessels not sharply defined 3. Fair vessel delineation; useful for diagnosis 4. Good vessel definition; small vessels visible 5. Excellent visualization of proximal through small distal vessels
*Overall imaging quality score* 1. Desired anatomy/features not seen 2. Unacceptable quality 3. Limited quality 4. Adequate quality 5. Higher than needed quality	

The arterial, capillary, and venous phases of each sDSA were assigned a score from 1 to 5 using the Subjective Imaging Grading Scale as adapted from [4]. Following this, an overall Imaging Quality Score from 1 to 5 was also assigned describing the overall quality of the entire sDSA as adapted from [18]. Possible scores for each sDSA ranged from 4 to 20. *sDSA* Synthetic digital subtraction angiography

Three board-certified neurointerventionalists evaluated each image series and provided a score from 1 to 5 for each of the arterial, capillary, and venous phases using the Söderman’s grading scale [4], as well as graded the overall sDSA series using the Image Quality Scoring Criteria subjective score from 1 to 5 [18]. The reader study provided each sDSA with a single score that could range from 4 (uninterpretable with no desired anatomy or features seen) to 20 (perfect reproduction of the arterial, capillary, and venous phases and the image provides higher than needed quality). The exact definition of the grading scale is summarized in Table 1. Kendall’s coefficient of concordance was calculated for each DSA study to determine the agreement between the reading neuroradiologists. Kendall’s *W* and *p* values were reported.

## Results

### Clinical data

Thirty-eight patients undergoing angiography for refractory cerebral vasospasm met the specified inclusion criteria and were identified. The mean cumulative radiation dosages for these 38 patients were 1,361.9 mGy per procedure. Of these 38 patients, 7 patient studies were excluded: 4 for significant motion artifact during diagnostic or post-therapeutic run, 2 for differing views obtained for pre- and post-verapamil studies, and 1 for different image magnifications obtained for pre- and post-verapamil studies. We identified the critical diagnostic and post-therapeutic runs and performed the experimental FRR and error measurements for these studies. Of the 31 patients that met the criteria for inclusion in the study, a random subset of 24 patients had their anteroposterior and lateral sDSA pairs evaluated in the reader study.

### Synthetic DSA

**Fig. 2:**
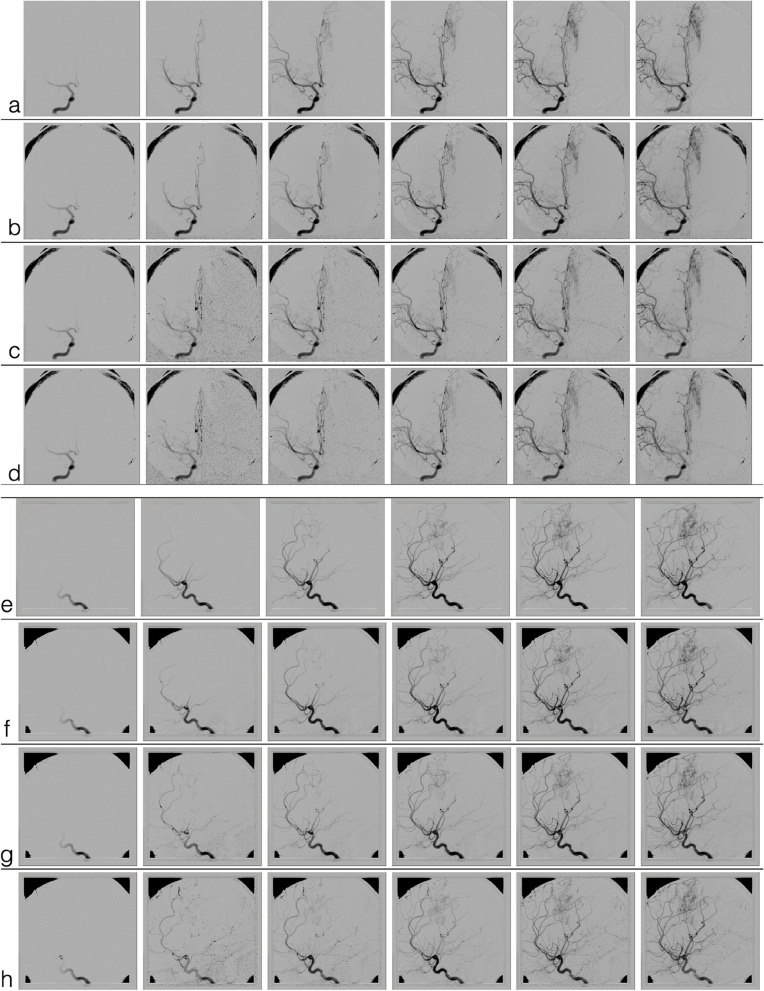
Comparison of original DSA (**a**, **e**) to 50% FRR sDSA (**b**, **f**), 66.6% FRR sDSA (**c**, **g**), and 75% FRR (**d**, **h**) sDSA. *DSA* Digital subtraction angiogram, *FRR *Frame rate reduction, *sDSA* Synthetic DSA

Figure 2 provides a subset of frames from a randomly chosen sDSA. In this representative figure, a right internal carotid artery injection was performed on a patient who was found to have a left-sided vascular malformation along the anterior cerebral artery territory. The row of frames without colored outline for each set of images is the index (ground truth) angiogram. The outlined frames represent synthetic angiograms generated from the subset of data following experimental FRR. A frame with a red outline indicates a captured frame acquisition, and the frame with a blue outline represents the synthetic reconstruction following a gamma variate model fit to the subset of data. Each sDSA took on average 0.51 s to generate.

### Model fit

**Fig. 3:**
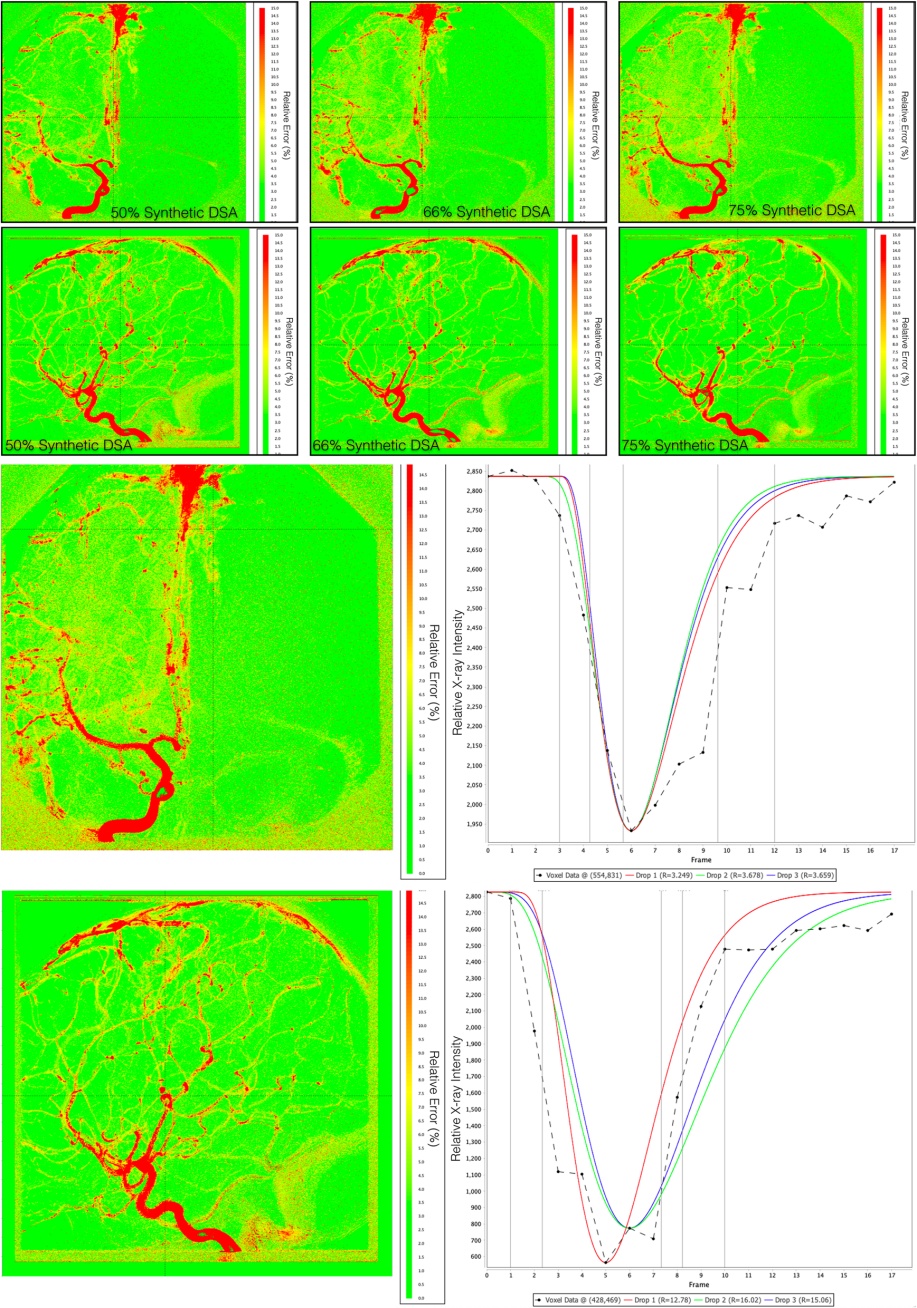
**a** Heatmaps demonstrating the percent error of the relative x-ray intensities at each voxel over the entire study when comparing the original angiographic data to the synthetically reconstructed study. Red represents ~ 15% relative x-ray intensity error and green representing < 3% error. From left to right, the error maps of a single study for each level of subtraction are presented. **b** Our system allows users to inspect every voxel of the error map on the left and demonstrates the gamma variate function fits for each level of frame rate reduction on the right. Drop 1, 2, and 3 represent 50%, 66%, and 75% frame reduction respectively. R is the percent error

Heatmaps of percent errors were generated for each sDSA study at each level of FRR (Fig. 3a). Errors were found to be lower in the brain parenchyma when compared to large diameter vasculature. sDSA anteroposterior view studies at 50%, 66%, and 75% reductions demonstrated an average voxel error of 2 ± 2.5%, 6.5 ± 1.5%, and 27.0 ± 2.0%, respectively. sDSA in lateral views at 50%, 66%, and 75% reductions demonstrated an average voxel error of 2.0 ± 2.2%, 15.0 ± 3.1%, and 14.8 ± 13.0%, respectively. Figure 3b demonstrates a snapshot of our perfusion analysis software and representative GV model fits at the three experimental levels of frame rate reduction.

### Reader study

Results of the reader study demonstrated an average rating of 12.8 (95% CI 12.3, 13.3) for the 75% sDSA, 12.7 (12.2, 13.2) for the 66% sDSA, and 12.0 (11.5, 12.5) for the 50% sDSA when evaluating the study based on the criteria outlined in Table 1. No statistically significant difference was found between sDSAs at varying degrees of FRR (Table 2). Kendall concordance test shows moderate concordance between the readers for all dose-reduced image sets with coefficients 0.491 (*p* = 0.019), 0.442 (*p* = 0.066), and 0.585 (*p* = 0.001) for 75%, 66%, and 50% synthetic frames, respectively. The overall coefficient of variation (defined as 100 * standard deviation/mean) was 26.1. Kendall’s coefficient of concordance for the scoring across different levels of FRR resulted into a *W* of 0.506.

**Table 2:** Summary of scores from the reader study by frame rate reduction

FRR percentage and readers	Number of graded studies	Score		
		Mean (SD)	Range	95% CI
75% FRR				
Reader 1	48	11.5 (3.6)	5.0–18.0	10.5, 12.5
Reader 2	48	12.9 (2.7)	6.3–19.0	12.1, 13.7
Reader 3	48	13.9 (2.2)	8.0–18.0	13.2, 14.5
Total	144	12.8 (3.0)	5.0–19.0	12.3, 13.3
66% FRR				
Reader 1	48	11.9 (3.9)	5.0–19.0	10.8, 13.1
Reader 2	48	12.2 (2.6)	6.3–19.3	11.5, 13.0
Reader 3	48	13.9 (2.2)	7.3–17.7	13.3, 14.5
Total	144	12.7 (3.1)	5.0–19.3	12.2, 13.2
50% FRR				
Reader 1	48	11.2 (3.5)	5.0–19.0	10.2, 12.2
Reader 2	48	11.4 (2.6)	6.0–18.7	10.6, 12.1
Reader 3	48	13.5 (2.0)	10.0–17.0	12.9, 14.0
Total	144	12.0 (2.9)	5.0–19.0	11.5, 12.5

Each synthetic DSA directly compared to the original DSA using the criteria shown in Table 1. *CI* Confidence interval, *DSA* Digital subtraction angiography, *FRR* Frame rate reduction, *SD* Standard deviation

## Discussion

Our findings of low objective error of synthesized frames, the grading of all sDSA studies as “adequate” and the concordance during the reader study imply that DSA acquisition rates can be lowered by 75% with preservation of diagnostic utility. This suggests that there may be an inherent redundancy in clinical DSA acquisition protocols and supports our hypothesis that performing a frame-to-frame interpolation with reduced acquisition rates can be a technique used to reduce radiation dosages. We found moderate concordance between readers assessing the clinical utility of the sDSAs at all levels of FRR. Concordance decreased with greater FRR, likely as a result of higher degrees of noise with greater frame rate reductions.

This technique of sDSA reconstruction is unique in that only frame rates were adjusted to provide a desired reduction in radiation. This allows for its modular application in conjunction with other radiation reduction techniques, presenting the potential for synergistic effects on radiation reduction [3–5]. The application of sDSAs in the clinical setting would reduce the overall radiation burden for patients who require surveillance imaging. Young patients with known cerebral aneurysms, arteriovenous malformations, or MoyaMoya disease would benefit from the reduced lifetime radiation dosage [19–22].

There are various limitations to this study and its applicability to different pathologies. First, the limited sample size and retrospective design present inherent limitations to this work with an impact on both the automated analysis and formal reader study. Second, some pathologies may not be well suited for evaluation by sDSAs, namely shunting lesions such as micro-arteriovenous malformations, dural arteriovenous fistulas, other small distal-branch vascular pathologies, and scenarios where significant motion precludes angiograms without motion, such as cardiac or chest angiography. Third, in this study, we did not specifically account for the contribution of recirculation after the first pass, extravascular diffusion of contrast when the blood–brain barrier is disrupted, or the overlap of vessels inherent to DSA [16, 23]. Future work will focus on improving contrast traversal modeling to better account for these different anatomic and pathologic circumstances.

While our findings indicate that an FRR of 75% can be achieved with this method with retention of diagnostic utility, an empirical minimum frame rate cannot be ascertained from our study. As some DSA series in the study had only 16 frames, further FRR beyond 75% would not provide enough remaining frames to fit the gamma variate functions. Further work is needed to evaluate sDSA’s sensitivity to noise such as motion artifact or decreased signal-to-noise ratio present at higher magnification rates, and inherent error with distal branch reconstruction. The degree of error introduced into sDSA with respect to these limiting factors was not evaluated.

One extension of this work can be the incorporation of a variable frame rate acquisition with selective under-sampling of contrast traversal phases that are not of interest [3]. Furthermore, we would like to quantify the errors based on vessel caliber utilizing kurtosis to evaluate sharpness as a function of FRR. Future investigation may also include a real-time comparison of this technique to control reference DSA. Other pathologies of interest such as sickle cell, MoyaMoya, arteriovenous malformations, hereditary hemorrhagic telangiectasia, and related vascular malformations may be evaluated with the specific goal of identifying empirical minimum frame rate may be established. Lastly, the generalizability of our method lends itself well to a similar investigation of radiation dose reduction in other angiographic modalities such as CT perfusion.

As presented, this work supports the potential benefit for algorithmic DSA FRR by as much as 75% and a proportional radiation reduction for the evaluation of, or follow-up for, specific pathologies, especially within the most vulnerable patient populations.

### Supplementary Information

**Additional file 1.**

## Data Availability

The datasets used and/or analyzed during the current study are available from the corresponding author on reasonable request.

## Abbreviations

CT: Computed tomography
DSA: Digital subtraction angiography
FRR: Frame rate reduction
GV: Gamma variate
MR: Magnetic resonance
RD: Radiation dosage
sDSA: Synthetic DSA

## Footnotes

1 See the Additional file 1 for Eqs. 2 and 3.
