# Supplementary material for: Synthetic interpolated DSA for radiation exposure reduction via gamma variate contrast flow modeling: a retrospective cohort study
Source: Eur Radiol Exp. 2024 Feb 16;8:25. doi: 10.1186/s41747-023-00404-2 (PMC10869670; doi:10.1186/s41747-023-00404-2)
Supplement: Supplementary file 1 — Additional file 1. [file 41747_2023_404_MOESM1_ESM.docx]

**Synthetic interpolated DSA for radiation exposure reduction via gamma variate contrast flow modeling: a retrospective cohort study**

**APPENDIX**

**Model fitting**

Due to the proportionality of the signal intensity ($I_{x,y}$) of a given voxel at location $(x,y)$ and time $(t)$ to the contrast volume ($C_{x,y}$) at the same location and time, intensity-time curves were fit using a GV model to characterize the flow of contrast through tissue(Bateman and Kruger 1984; Scalzo and Liebeskind 2016; Abumoussa et al. 2020).

The GV function is mathematically defined as:

$I_{x,y}\left( t \right)=A{(t-AT)}^{\alpha}ⅇ^{\frac{-(t-AT)}{\beta}}$ [Equation 2]

Here, $A$ is a scaling factor; $AT$ is the arrival time; $\alpha$ represents the degree of turbulence of flow; and $\beta$ carries the physiological significance of the volume of the theoretical mixing chamber to the flow rate.

Equation [2] explicitly expresses signal intensity as a function of time and as such is appropriate for synthesizing intervening data points at an arbitrary time $t$. However, this form is not computationally efficient for multivariate curve fitting and extraction of free parameters $\alpha$, $\beta$, and $A$. Therefore, an alternate form with decoupled parameters amenable to linear regression is substituted (Madsen 1992) to allow for an optimized numerical analsyis:

$\ln\left( I_{x,y}\left( t^{'} \right) \right)=\ln\left( I_{max} \right)+\alpha(1+\ln\left( t^{'} \right)-t^{'})$ [3]

Where $t^{'}=\frac{t-AT}{t_{max}-AT}$ and $t_{max}$ is the time at which the signal intensity is at a maximum ($I_{max}$). Our approach uses equation [3] to perform linear least squares fit with $\ln\left( I_{x,y}\left( t^{'} \right) \right)$ and $\left( 1+\ln\left( t^{'} \right)-t^{'} \right)$ as the variables. $\alpha$ is then yielded as the slope of this linear regression, $\beta$ is expressible as $\frac{t_{max}}{\alpha}$, and $A$ can be calculated as $\frac{1}{I_{\max}}{(t_{\max}-AT)}^{\alpha}ⅇ^{\frac{-(t_{\max}-AT)}{\beta}}$. Before performing this linear regression, however, data is transformed to $t^{'}$, in terms of the temporal parameters $t_{max}$ and $AT$. These parameters are deduced in the following order and are summarized in Fig. 1e,f:

1. $AT$ is identified as the point where the signal intensity curve rises to 10% of the range for the specific voxel. If no single point is found, $AT$ is calculated as the time at which there is 10% rise from the minimum intensity value ${(I}_{min}$) of a linear interpolant between $I_{min}$ and $I_{max}$.
2. Time to peak ${(t}_{max})$ is identified as the time point where the intensity-time curve is at $I_{max}$. The accuracy of capturing the true $t_{max}$, is dependent of the sampling rate of an angiogram. To prevent such errors, we used the intersection of two lines involving the 4 points about $I_{max}$. The first line is drawn between $I_{max}$ and the point before it while the second line is drawn between the two points after $I_{max}$. $t_{max}$ is defined to be the time at which these two lines intersect.
3. Leave time $(LT)$ is identified as the first inflection point after $t_{max}$ when the recirculation volume can be expected to dominate the signal curve. If no value is observed that satisfies this condition, $LT$ is calculated as $AT+2(t_{max}-AT)$. $LT$ is the latest datapoint considered in the time series for the subsequent regression for parameter extraction.
